# Improved syntheses of high hole mobility phthalocyanines: A case of steric assistance in the cyclo-oligomerisation of phthalonitriles

**DOI:** 10.3762/bjoc.8.14

**Published:** 2012-01-24

**Authors:** Daniel J Tate, Rémi Anémian, Richard J Bushby, Suwat Nanan, Stuart L Warriner, and Benjamin J Whitaker

**Affiliations:** 1 Centre for Molecular Nanoscience (CMNS), University of Leeds, Woodhouse Lane, Leeds, LS2 9JT, United Kingdom; 2 Merck KGaA, Industriepark Höchst, D 65926 Frankfurt am Main, Germany; 3 School of Chemistry, University of Leeds, Woodhouse Lane, Leeds, LS2 9JT, United Kingdom

**Keywords:** high hole mobility, phthalocyanine, steric assistance, Thorpe–Ingold effect, time-of-flight

## Abstract

It has been shown that the base-initiated cyclo-oligomerisation of phthalonitriles is favoured by bulky α-substituents making it possible to obtain the metal-free phthalocyanine directly and in high yield. The phthalocyanine with eight α-isoheptyl substituents gives a high time-of-flight hole mobility of 0.14 cm^2^·V^−1^·s^−1^ within the temperature range of the columnar hexagonal phase, that is 169–189 °C.

## Introduction

Liquid crystalline semiconductors [1–2] are potentially useful in the fabrication of organic field-effect transistors [3–4], light-emitting devices [5–7] and photovoltaic devices [8–11]. Most of the interest has centred on using them as hole conductors and a lot of effort has been expended on designing columnar liquid crystals with high hole mobilities. There are fewer examples of good liquid crystalline electron conductors, and most of those known can only be used in an oxygen-free environment [12–13]. However, the columnar phases of 1,4,8,11,15,18,22,25-octaoctylphthalocyanine show good time-of-flight transits for both holes and electrons, together with exceptionally high mobilities (time-of-flight hole mobilities of 0.20 cm^2^·V^−1^·s^−1^ in the Col_r_ phase at 85 °C and 0.10 cm^2^·V^−1^·s^−1^ in the Col_h_ phase at 100 °C; electron mobilities of 0.30 cm^2^·V^−1^·s^−1^ in the Col_r_ phase at 85 °C and 0.20 cm^2^·V^−1^·s^−1^ in the Col_h_ phase at 100 °C) [14–15]. Furthermore, this phthalocyanine gives good time-of-flight electron transits even in an ambient atmosphere [15]. As a result, related α-alkylated phthalocyanines are attracting interest for use in organic devices, such as solar cells [16], and this stimulated our efforts to produce further examples [17]. As part of that work we investigated cases in which the α-substituents were branched-chain types. Since we did not want to produce mixtures of diasterioisomers, we used either citronellol-based chains (enantiomerically pure substrates from the chiral pool) or symmetrical, nonchiral R_2_CH-terminated chains, which we synthesised from commercially available carboxylic acids or alcohols. We discovered that bulky, branched-chain α-substituents provide steric assistance in the conversion of the phthalonitrile precursors to the phthalocyanines, thus leading to substantially enhanced yields. Just as the cyclo-oligomerisation reaction is favoured by the “pull” of a transition metal template, it can also be enhanced using the “push” of suitable α-substituents. This makes the metal-free phthalocyanines much easier to produce on a multigram scale.

## Results and Discussion

### Synthesis

**Scheme 1:**
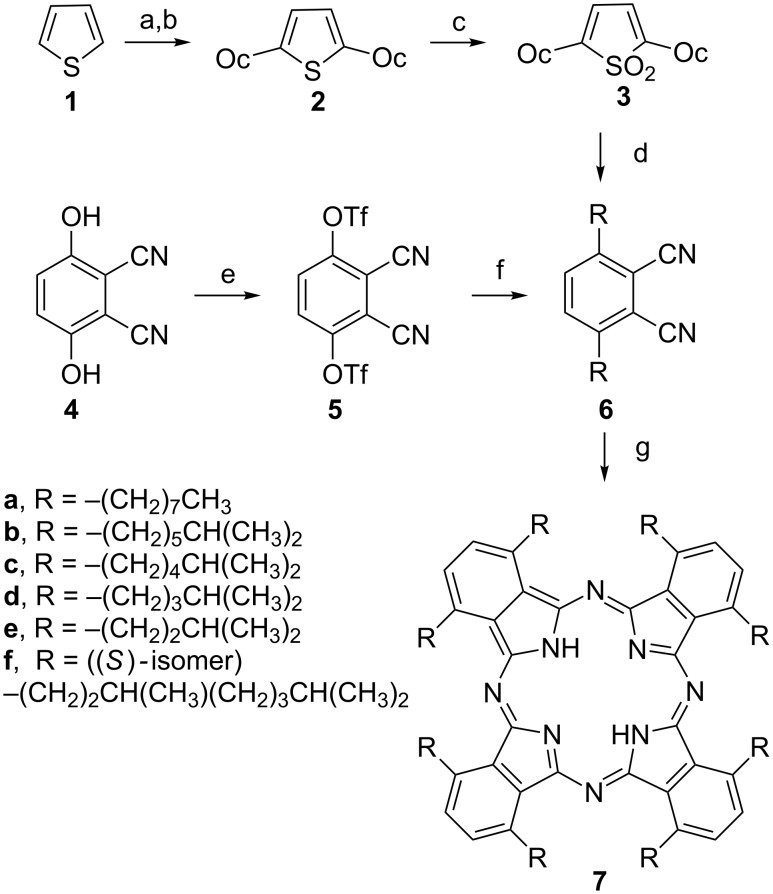
(Top) original synthesis of compound **7a** from thiophene (Oc = *n*-octyl) [19]. (Below) shortened synthesis of α-alkylated derivatives through the triflate of 3,6-dihydroxyphthalonitrile. Reagents: (a) BuLi; (b) C_8_H_15_Br; (c) MCPBA , 35–51%; (d) fumaronitrile/Δ, 40–48%; (e) (Tf)_2_O; (f) (i) RZnI, NiCl_2_(PPh_3_)_2_, PPh_3_, BuLi (ii) KBr/−78 °C; (g) (i) Li/C_5_H_11_OH/Δ, (ii) AcOH.

In synthesising many different α-alkylated phthalocyanines [17] we experimented with various routes to the phthalonitrile precursors **6** (Scheme 1). The route that we originally used was one described by Cook et al. for the octaoctyl compound **7a** [18]. This involved bis-alkylation of thiophene (**1**), oxidation to the corresponding sulfone **3**, and treatment with fumaronitrile (Scheme 1, top line). However, for most of the phthalonitriles we have made, we have found that a much better route is the nickel-catalysed reaction of an alkylzinc iodide with the bistriflate of 3,6-dihydroxyphthalonitrile **5** (Scheme 1, middle line), previously described in the patent literature [19–20].

**Scheme 2:**
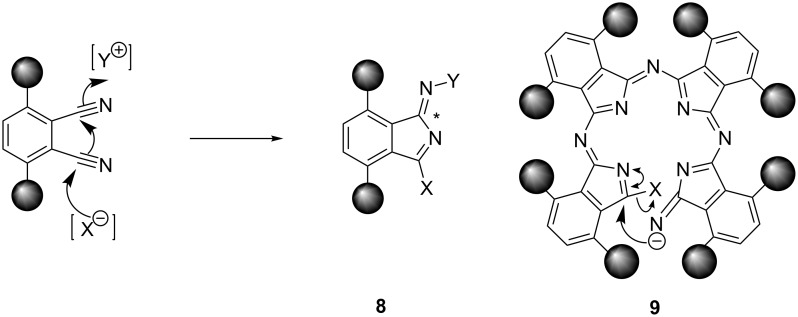
Effect of bulky α-alkyl substituents on the base-initiated cyclo-oligomerisation of phthalonitriles.

The phthalonitriles made in this way were converted to the phthalocyanines by treating them with lithium pentoxide in refluxing pentanol [18,21–32]. Yields of phthalocyanines prepared by this route are generally poor and usually less than 25% [29–32]. This, together with the need for chromatographic purification, limits the scale on which metal-free phthalocyanine liquid crystals can be made. Such low yields are common for nontemplated phthalonitrile cyclo-oligomerisation reactions and, although high-yielding nontemplated routes have been developed for some metal-free phthalocyanines [19,33–35], for the liquid-crystalline phthalocyanines, the low yield of the cyclo-oligomerisation step is a problem. Somewhat surprisingly, for some of the branched-chain α-alkylated systems, we obtained very good yields even without using a metal template. As shown in Table 1, not only are some of these yields exceptionally high (for nontemplated phthalonitrile oligomerisation), but there is also a clear relationship between the isolated, recrystallised yield and the steric bulk of the α-substituent. As the bulk of the side-chain is increased or as the branch point in the side-chain is moved closer to the nucleus, so the yield improves. In order to check that the high yields were not the result of templating by adventitious transition metal ion impurities, the phthalocyanine **7f** was subjected to ICP-MS trace metal impurity analysis, but levels of transition metal impurities were found to be below the IPC-MS detection limit (ppb). The most likely explanation for the effect of these branched-chain substituents on the yields of these reactions is shown schematically in Scheme 2. In the intermediates **8** (Scheme 2, X = C_5_H_11_O, Y = growing oligomer chain) the exocyclic carbon/nitrogen double bond marked * presumably has the (*Z*)-stereochemistry shown. This is the favoured stereochemistry for most imides of aromatic ketones, although the opposite stereochemisty is only a little bit less stable if the *ortho*-positions of the benzene ring are unsubstituted [36]. On the other hand, if these “*ortho*”-positions are substituted, this should provide a bias towards the (*Z*)-isomer. More significant in the context of phthalocyanine formation is the cumulative effect of these substituents on the conformation of the open-chain tetrameric precursor **9** (or its equivalent, Scheme 2) to the phthalocyanine nucleus. Examination of models for compound **9** shows that it is impossible for this intermediate to achieve total planarity. More important is the fact that an increase in the bulk of the alkyl substituents in the “*ortho*” positions will reinforce the preference for a (*Z*)-configuration about all of the exocyclic carbon/nitrogen partial double bonds, all six of which need to be *Z* in the cyclisation transition state. Hence, since the substituents would restrict the conformational space available to the intermediate, they would favour cyclisation. This phenomenon is clearly related to the effect of *gem*-dimethyl groups on the cyclisation of acyclic compounds (the Thorpe–Ingold effect) [37]. From the standpoint of making phthlocyanine-based liquid crystals, its importance is that it provides a simple, high-yielding route to metal-free α-alkylated phthalocyanines.

**Table 1:** Isolated yields of purified phthalocyanine (chromatographed, and in the case of **7a**–**7e** crystallised) obtained by treating the corresponding phthalonitrile **6** for ca. 16 h with lithium pentoxide in refluxing pentanol. For α-alkylated phthalocyanines with *n*-alkyl substituents, the average yield obtained by this route is 22 ± 5% (based on the fourteen known examples) [29–32].

	Alkyl chain	Yield (%)

**7a**	–(CH_2_)_7_CH_3_	17 [25]
**7b**	–(CH_2_)_5_CH(CH_3_)_2_	28
**7c**	–(CH_2_)_4_CH(CH_3_)_2_	45
**7d**	–(CH_2_)_3_CH(CH_3_)_2_	62
**7e**	–(CH_2_)_2_CH(CH_3_)_2_	78
**7f**	((*S*)-isomer) –(CH_2_)_2_CH(CH_3_)(CH_2_)_2_CH(CH_3_)_2_	83

### Liquid-crystal properties

**Figure 1:**
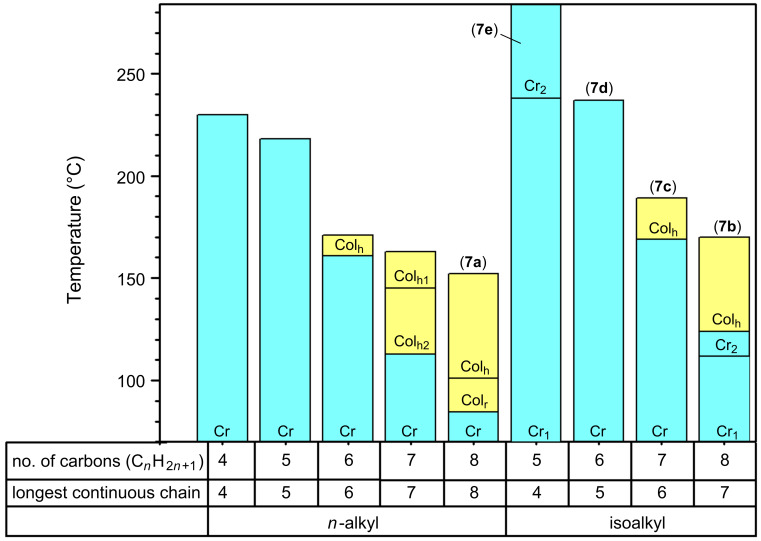
Comparison of the phase behaviours of the α-substituted phthalocyanines with *n*-alkyl [25] and isoalkyl side chains. The stable ranges for the crystal phases are shown in blue and those of the columnar liquid-crystalline phases in yellow.

**Figure 2:**
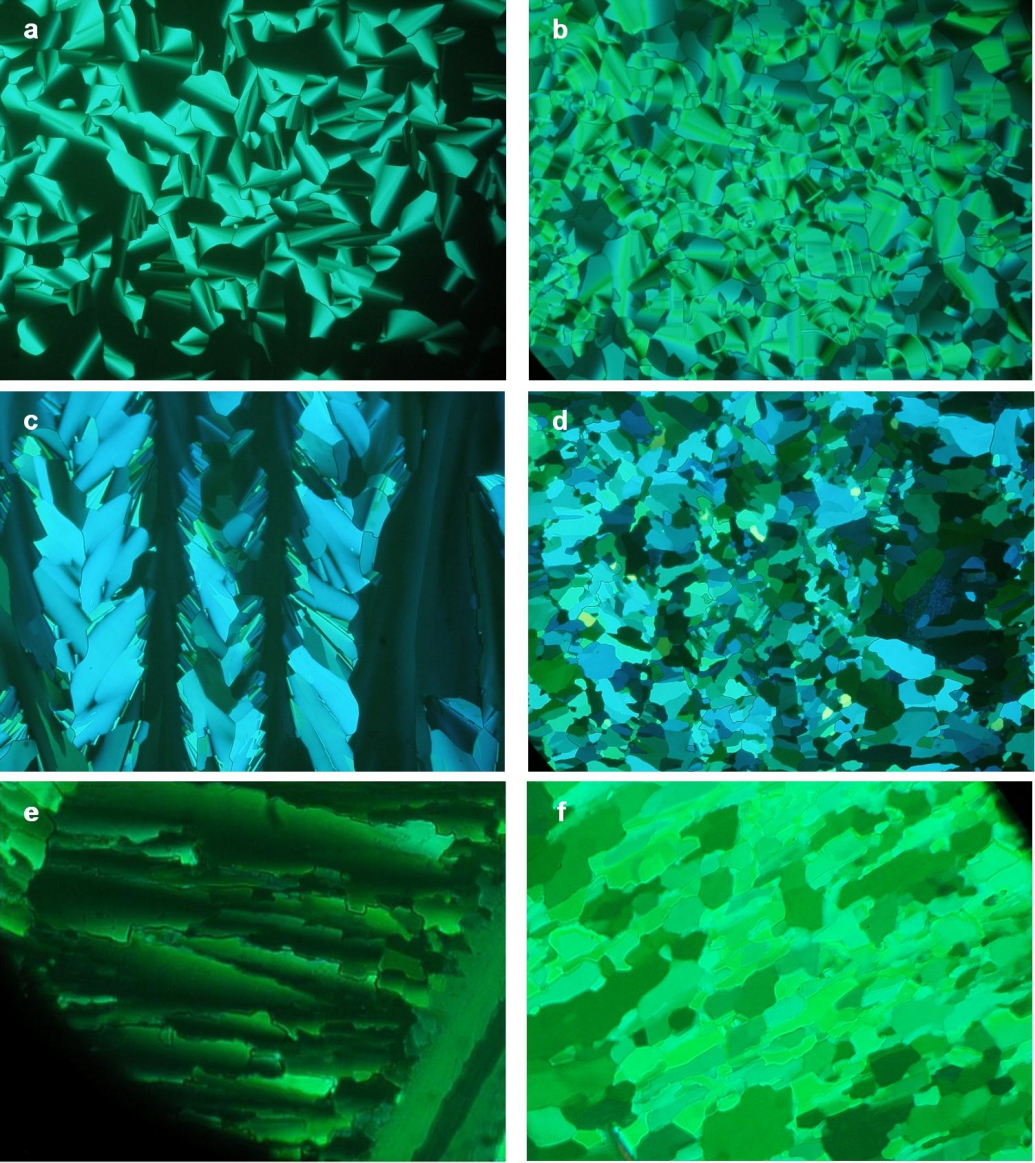
Optical micrographs taken with crossed polarisers and at a magnification of × 20. (a) *n*-Octyl derivative **7a** in the Col_h_ phase at 145 °C. (b) **7a** in the Col_r_ phase at 100 °C. (c) Isooctyl derivative **7b** in the Col_h_ phase at 170 °C. (d) **7b** in the unknown (but probably Cr) phase at 120 °C. (e) Isoheptyl derivative **7c** in the Col_h_ phase at 170 °C. (f) **7c** in the Cr phase at 155 °C.

The liquid-crystal behaviour of the compounds we synthesised was investigated by polarising microscopy, differential scanning calorimetry (DSC) and, in the cases of compounds **7c–7e**, by low-angle X-ray diffraction. The DSC data is summarised in Table 2, the phase behaviour of the isoalkyl series is compared graphically with that for the *n*-alkyl series in Figure 1 and typical polarizing micrographs are shown in Figure 2. Compounds **7b** and **7c** exhibit Col_h_ columnar mesophases. For compound **7b** the nature of the phase formed at temperatures below that for the Col_h_ phase (the phase formed between 112 and 124 °C) remains uncertain, but comparison of the optical textures (compare Figure 2d with Figure 2b and Figure 2f) suggests that it is crystalline rather than liquid-crystalline, and its texture is clearly very different to that of the Col_r_ phase of **7a** (Figure 2b). The X-ray data obtained for compound **7c** in the Col_h_ phase at 170–195 °C shows a 110/200 diffraction band corresponding to *a* = 19.2 Å and a broad band corresponding to a *d* spacing of 3.5–5.0 Å. For compound **7a** in its Col_h_ phase, Cook et al. reported a 110/200 band *a* = 22.6 Å, a very weak 310 band at ~13.0 Å, and a broad band corresponding to a *d* spacing of 3.5–5.0 Å [30]. In our case we were not able to detect the weak 310 band, but this is not unusual for the Col_h_ phase. The X-ray diffraction experiments confirmed that the phases formed by **7c** below 169 °C were crystalline and not columnar liquid-crystalline (not Col_r_) in nature and that compounds **7d** and **7e** did not give liquid-crystalline phases.

**Table 2:** Phase behaviour of the phthalocyanines determined by differential scanning calorimetry (second heating and first cooling cycle and a heating/cooling rate of 10 °C min^−1^).

	Alkyl chain	Phase behaviour °C (ΔH, J·g^−1^)

**7b**	–(CH_2_)_5_CH(CH_3_)_2_	Cr_1_ 112 (15) Cr_2_ (?)124 (9) Col_h_ 170 (11) I 162 (−11) Col_h_ 105 (−9) Cr_2_ (?) 20 (−9) Cr_1_
**7c**	–(CH_2_)_4_CH(CH_3_)_2_	Cr 169 (14) Col_h_ 189 (35) I 169 (−17) Col_h_161 (−14) Cr_1_ 140 (−11) Cr
**7d**	–(CH_2_)_3_CH(CH_3_)_2_	Cr 237 (43) I 223 (−51) Cr
**7e**	–(CH_2_)_2_CH(CH_3_)_2_	Cr_1_ 238 (2) Cr_2_ 284 (40) I 190 (−43) Cr_1_
**7f**	((*S*)-isomer) –(CH_2_)_2_CH(CH_3_)(CH_2_)_2_CH(CH_3_)_2_	Isotropic liquid at rt

Because suitable precursors for most isoalkyl chains are not commercially available and because (at the simplest level of theory) the space-filling properties of disordered/fluid *n-*alkyl and isoalkyl chains are expected to be “much the same”, there have been few studies of the differences between the liquid-crystal properties of *n-*alkyl and isoalkyl-substituted liquid-crystal derivatives. However, Gray and Kelly have shown that in the cyanobiphenyl and cyanoterphenyl series the differences are substantial with the N/I transition temperatures being lower in the isoalkyl series by between 8 and 50 °C (on the basis of comparing chains of equal maximum length): An effect reasonably attributed to the greater lateral separation between the molecules in the isoalkyl series [38]. In the isoalkyl-substituted phthalocyanines **7** the Col–I transition temperatures are a little higher than they are in the *n-*alkyl series (by 18 or 26 °C on the basis of a comparison of chains with the same number of carbon atoms; by 7 or 18 °C on the basis of a comparison of chains of maximum equal length). This may reflect a stronger column–column interaction within the Col_h_ phase, and it is consistent with the observation that the column–column spacing for **7c** (the isoheptyl derivative) is rather shorter than the value extrapolated for the *n-*hexyl derivative from the values reported for the *n-*heptyl and *n-*octyl compounds [30].

### Time-of-flight photoconductivity

**Figure 3:**
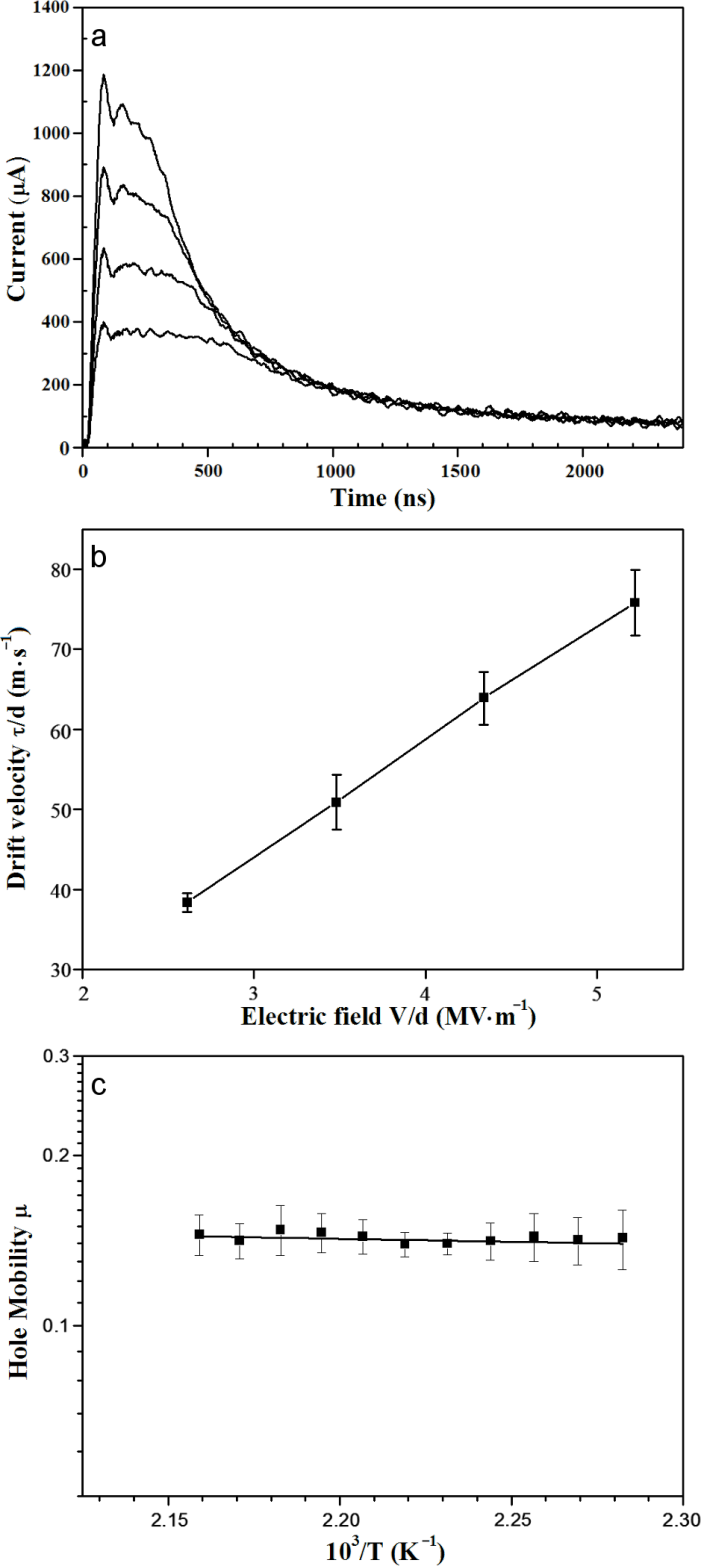
(a) Time-of-flight hole transits for an ITO/**7c**/ITO cell with the liquid crystal in its Col_h_ phase at 185 °C. Applied voltages 60 V (top trace), 80 V, 100 V, 120 V (bottom trace). (b) Drift velocity as a function of electric field (185 °C). (c) Temperature-dependence of the hole mobility within the Col_h_ phase. The error bars represent 2σ.

The hole mobility for compound **7c** was measured in its Col_h_ phase in the temperature range 169–189 °C. Slow cooling of a thin film sandwiched between ITO-coated glass slides readily gave the required homotropically aligned sample. However, a problem was encountered with the ToF measurements. Because the transit times are very short, the electronic noise generated by firing of the laser was found to overlap with the transient signal significantly, making it difficult to determine the transit times [39]. To overcome this problem, we found it necessary to increase the working distance between the sample and the laser. Even at the greatest practicable working distance (several metres), some noise was still seen in the first 200–300 ns of the transit, however, it was sufficiently reduced in the critical 300–800 ns region such that transit times could be determined. Figure 3a shows the time-of-flight signals for a sample of **7c** with a thickness of 23 μm at applied voltages of 60–120 V and at 185 °C. Figure 3b shows the plot of drift velocity as a function of field, each point being an average of five or more independent measurements. The mobilities were obtained from the slopes of these plots at each temperature. As shown in Figure 3c, the mobility is almost temperature-independent within the mesophase range. The hole mobility of **7c** in the Col_h_ phase at 185 °C was found to be 0.14 cm^2^·V^−1^·s^−1^, which is a little higher than that value previously reported for **7a** of 0.10 cm^2^·V^−1^·s^−1^ in the Col_h_ phase at 100 °C [14].

## Conclusion

Mobilities measured by the time-of-flight method are usually significantly lower than those measured by the pulse-radiolysis time-resolved microwave conductivity (PR-TRMC) method because of the very high frequency employed in PR-TRMC. At such high frequencies the charge carriers only migrate over microdomains, avoiding deep traps. Furthermore PR-TRMC records the combined mobilities of the holes and the electrons. Hence, although there are a number of PR-TRMC mobilities for liquid crystals that are as high as, or even higher than, that for **7c**, a value of 0.14 cm^2^·V^−1^·s^−1^ is one of the very highest recorded for a macrodomain (time-of-flight) hole mobility of a liquid crystal. The reason why the mobilities of these α-alkylated phthalocyanines are this high is not entirely easy to understand. Mobilities in liquid crystals are usually intermediate between those measured in crystalline and amorphous organic solids, and the mobility is primarily related to the degree of positional [40–44] or charge [45–46] disorder within the system. In most cases where a high time-of-flight mobility has been reported, the observation can be related to exceptionally high ordering in the mesophase, as revealed by X-ray diffraction (as in the Col_p_ phases of hexakispropyloxytriphenylene and hexakisbutyloxytriphenylene, the H phase of hexakishexylthiotriphenylene and the Col phases of CPI discotics) [40,42]. In the case of these α-alkylated phthalocyanines, there is no evidence from the X-ray studies of any particular “higher” order in the mesophase but, in comparing them to the triphenylenes, it is important to take into account the larger size of the aromatic core [47].

The steric assistance provided to the base-initiated cyclisation of α-alkylated phthalonitriles, when the α-substituents are branched-chain, enables the corresponding metal-free phthalocyanines to be made easily and on a multigram scale. Although in the examples given in this paper the compounds formed in the highest yields (for those phthalonitriles branched at the 3-position of the alkyl chain) are not liquid-crystalline, liquid-crystalline examples with branching at the 3-position could easily be designed.

## Experimental

### DSC Studies

DSC studies used a Perkin Elmer DSC-7 instrument calibrated with indium (mp 156.60, ΔH = 28.53 J·g^−1^) and a heating/cooling rate of 10 °C·min^−1^.

### Time-of-flight photoconductivity studies

The cells were assembled in a laminar-flow hood to avoid contamination by dust or grease. Each ITO-coated glass slide was connected to a copper wire by epoxy resin (Aradite) and the tip of the wire was bonded to the ITO surface with silver paint (RS 186-3600). The two halves were separated by a PET spacer (Goodfellow Cambridge Limited) with a thickness of 23 μm, held in position with clips and secured with epoxy resin. The thickness of the cell (d) was accurately determined by measuring the UV–vis scattering spectrum of the empty cell. The liquid-crystal sample was filled into the cell in its isotropic phase by capillary action. The sample was heated up to a few degrees above the liquid-crystal to isotropic phase transition temperature and then was cooled into the liquid-crystal phase at 0.1 °C·min^−1^ to produce a monodomain, homotropically aligned sample (checked by polarising microscopy). The temperature of the cell was then controlled ± 0.1 °C on a Linkam hot stage. A field of 2.5–5.5 MV·m^−1^ (applied voltage, V ~ 60–120 V) was applied across the cell, a laser pulse (Nd-YAG laser, wavelength of 355 nm, intensity 30 mJ/pulse before filtering) was used to illuminate one side, and the resultant transits were recorded. Calculations show that this light is absorbed within <1 μm of the electrode surface. In some cases, a neutral density filter was used to reduce the laser power. The transit times τ were determined from the inflection point in a double logarithmic plot of the measured transient photocurrents, from a series of at least five independent experiments. The electron mobility for **7c** was not determined since the inflection in the time-of-flight signal was insufficiently distinct. As in the case of **7a**, and indeed as in the case of all other “high mobility” liquid crystals, there was no evidence for field dependence of the mobility. Hence, the mobility μ (= d^2^/Vτ) was determined from the slope of a linear fit to the plot of drift velocity (τ/d) versus field (V/d), as shown in the example in Figure 3b.

### Synthesis

#### General procedures

Flash chromatography was carried out on Merck (230–400 mesh) silica with HPLC grade solvent as eluent. TLC was carried out on Merck silica gel (60 Å) F_254_ coated glass plates. Melting points were recorded on a Linkam LTSE300 heating stage. IR spectra were carried out on a Perkin Elmer Spectrum 1 FT-IR spectrometer. ^1^H and ^13^C{^1^H} NMR spectra were recorded on a Bruker DPX300 spectrometer. Proton and carbon signals were assigned by using a combination of ^1^H/^1^H (COSY) and ^13^C{^1^H}/^1^H (HMBC and HMQC} correlation methods. Both EI and FD mass spectra were recorded on a Waters CGT micromass instrument. ES mass spectra were recorded on a Bruker Datonics Micro ToF instrument. Elemental analyses were performed in the Microanalysis Department of the School of Chemistry, University of Leeds.

#### 3-Methylbutyl iodide [48]

To a flame-dried flask purged with argon was added triphenylphosphine (44.61 g, 0.170 mol), imidazole (16.16 g, 0.255 mol) and anhydrous diethyl ether/acetonitrile (125 mL/125 mL). The stirred reaction mixture was cooled (0 °C, ice bath) and 3-methylbutanol (7.50 g, 0.085 mol) was added. After 10 min, iodine (28.07 g, 0.111 mol) was added in portions. Upon completion of the addition the mixture was allowed to warm to rt and stirred for a further 2 h. The mixture was diluted with pentane (200 mL), and washed sequentially with sat Na_2_S_2_O_3_ (2 × 50 mL), sat CuSO_4_ (2 × 50 mL), brine (50 mL), dried (MgSO_4_) and concentrated in vacuo. The crude product was purified on silica gel with pentane as eluent (*R*_f_ ~0.70) to afford the title compound as a homogeneous oil (15.3 g, 91%).

IR (neat): 2956 (C–H), 2929 (C–H), 2906 (C–H), 2870 (C–H) cm^−1^; ^1^H NMR (CDCl_3_, 300 MHz) δ 3.21 (t, *J* = 7.2 Hz, 2H, (CH_3_)_2_CHCH_2_*CH*_2_I), 1.74–1.69 (m, 3H, (CH_3_)_2_*CHCH*_2_CH_2_I), 0.91 (d, *J* = 6.37 Hz, 6H, (*CH*_3_)_2_CH(CH_2_)_2_I); ^13^C NMR (CDCl_3_, 75 MHz) δ 42.6 (C2), 29.1 (C3), 21.7 (C4), 5.4 (C1); HRMS–EI (70 eV) *m*/*z*: [M − I]^+^ calcd for C_5_H_11_, 71.0860; found, 71.0854; Anal. calcd for C_5_H_11_I: C, 30.23; H, 5.60; I, 64.08; found: C, 30.40; H, 5.70; I, 63.80.

4-Methylpentyl iodide [49], 5-methylhexyl iodide [50–51], 6-methylheptyl iodide and (*S*)-3,7-dimethyloctyl iodide [52] were obtained in the same manner (see Supporting Information File 1).

#### 3-Methylbutylzinc iodide

Activated zinc dust was always freshly prepared in the same glassware to be used for the subsequent stages. A suspension of zinc (7.85 g, 120 mmol) was stirred in a 2% solution of HCl (20 mL) for 2 min. The resulting dust was sequentially stirred and decanted with a 2% solution of HCl (20 mL), water (3 × 20 mL) and EtOH (2 × 20 mL). The resultant suspension was allowed to settle, decanted and washed with Et_2_O (20 mL). Finally the remainder of the glassware for the reaction was assembled and the activated zinc (ca. 6.54 g, 100 mmol) was dried under vacuum (ca. 0.1 mbar), purged with argon, flame dried under vacuum (ca. 0.05 mbar) and again purged with argon ready for use.

Under an argon atmosphere, the freshly prepared activated zinc dust (16 g, 0.23 mol, 3 equiv) was stirred in anhydrous THF (30 mL). Dibromoethane (0.97 mL, 0.011 mol, 15 mol %) was added via syringe and the mixture was heated under reflux for ca. 10 min, cooled, and again heated under reflux for ca. 10 min and cooled. Trimethylsilyl chloride (1.44 mL, 0.011 mol, 15 mol %) was added and the mixture heated under reflux once more and allowed to cool. 3-Methybutyl iodide (12 g, 0.061 mol) was added via syringe and the mixture was stirred for 16 h at 40 °C. Upon completion of the reaction, the mixture was allowed to cool to rt and the excess zinc was allowed to settle. The resultant grey solution was transferred to a Schlenk tube via cannula, and the remaining zinc powder was washed with THF (30 mL) and transferred to the Schlenk tube to afford the title compound (62 mL, 0.90 M solution, assuming a ~90% conversion).

#### 3,6-Bis(trifluoromethanesulfonyloxy)phthalonitrile (**5**) [20]

To a flame-dried flask, trifluoromethanesulfonic anhydride (24.6 mL, 149 mmol) dissolved in anhydrous DCM (20 mL) was added dropwise to a cooled (−20 °C) stirred solution of 2,3-dicyanohydroquinone (**4**) (10.0 g, 62.5 mmol) dissolved in anhydrous DCM (30 mL) and 2,6-lutidine (60 mL). Upon completion of the addition, the reaction mixture was maintained at −20 °C for 1 h and then allowed to warm to rt and stirred for a further 16 h. The reaction was quenched by the addition of water (20 mL), extracted into EtOAc (3 × 50 mL), washed successively with 10% NaOH (2 × 30 mL), 10% HCl (2 × 30 mL), brine (2 × 20 mL), dried (Na_2_SO_4_) and concentrated in vacuo to afford the title compound as colourless prisms (18.8 g, 71%). mp (MeOH) 109–110 °C (lit [19] 109–111 °C); IR (neat): 2252 (CN), 1471 (C=C), 1436 (C=C), 1223 (S=O) cm^−1^; ^1^H NMR (CDCl_3_, 300 MHz) δ 7.87 (s, 2H, 4,5H); ^13^C NMR (CDCl_3_, 75 MHz) δ 148.9 (C3,6), 128.7 (C4,5), 114.6 (q, *J* = 319.5 Hz, 2 × CF_3_), CN not observed; HRMS–EI (70 eV) *m*/*z*: [M]^+^ calcd for C_10_H_2_N_2_S_2_F_6_O_6_, 423.9259; found, 423.9272; Anal. calcd for C_10_H_2_N_2_S_2_F_6_O_6_: C, 28.31; H, 0.48; N, 6.60; S, 15.12; found: C, 28.20; H, 0.35; N, 6.55; S, 15.10.

#### 3,6-Bis(3-methylbutyl)phthalonitrile (**6e**)

To a flame-dried flask, under an argon atmosphere, were added bis(triphenylphosphine)nickel(II) dichloride (1.18 g, 1.82 mmol), triphenylphosphine (0.95 g, 3.6 mmol) and anhydrous THF (40 mL). *n*-Butyllithium (1.45 mL, 3.64 mmol, 2.5 M in hexanes) was added to the stirred mixture to afford a blood red slurry. 3,6-Bis(trifluoromethanesulfonyloxy)-phthalonitrile (7.71 g, 18.2 mmol) and KBr (6.48 g, 54.5mmol) was added under a fast stream of argon. The resultant brown solution was cooled to −78 °C. Freshly prepared 3-methylbutylzinc iodide (62.4 mL, 56.17 mmol, 0.90 M solution in THF) was added dropwise over a period of 1 h via cannula from a Schlenk flask. Upon completion of the addition, the reaction mixture was allowed to warm to rt and stirred for a further 16 h. The reaction was quenched by the careful addition of 10% HCl (10 mL) and the mixture was extracted with EtOAc (3 × 30 mL). The combined organic extracts were successively washed with 10% HCl (20 mL), 5% NaOH (20 mL), brine (20 mL), dried (MgSO_4_) and concentrated in vacuo. The resultant yellow solid was purified by chromatography on silica gel with 5% EtOAc/hexane (v:v) as eluent until all triphenylphosphine was extracted. The title compound [24] was isolated as colourless needles (3.51 g, 72%) by increasing the polarity to 10% EtOAc/hexane (v:v). *R*_f_ ~ 0.30; mp (petroleum ether) 62–63 °C; IR (neat): 2957 (C–H), 2936 (C–H), 2872 (C–H), 2226 (CN), 1468 (C=C), 1459 (C=C) cm^−1^; ^1^H NMR (CDCl_3_, 500 MHz) δ 7.42 (s, 2H, C4H and C5H), 2.85 (t, *J* = 8.1 Hz, 4H, Ar*CH*_2_CH_2_CH(CH_3_)_2_), 1.64 (m, *J* = 6.5 Hz, 2H, Ar(CH_2_)_2_*CH*(CH_3_)_2_), 1.54 (m, 4H, ArCH_2_*CH*_2_CH(CH_3_)_2_), 0.97 (d, *J* = 6.6 Hz, 12H, Ar(CH_2_)_2_CH(*CH*_3_)_2_); ^13^C NMR (CDCl_3_, 125 MHz) δ 146.44 (C3 and C6), 133.43 (C4 and C5), 115.60 and 115.11 (C1, C2 and CN), 39.91 (C'2), 32.38 (C'1), 27.87 (C'3), 22.33 (C′4); HRMS–EI (70 eV) *m*/*z*: [M−CH_3_]*^+^* calcd for C_17_H_21_N_2_, 253.1704; found, 253.1696 (100); Anal. calcd for C_18_H_24_N_2_: C, 80.55; H, 9.01; N, 10.44; found: C, 79.90; H, 8.95; N, 10.30.

3,6-Bis(4-methylpentyl)phthalonitrile (**6d**), 3,6-bis(5-methylhexyl)phthalonitrile (**6c**), 3,6-bis(6-methylheptyl)phthalonitrile (**6b**) and 3,6-bis((*S*)-3,7-dimethyloctyl)phthalonitrile (**6f**) were prepared in the same manner (see Supporting Information File 1).

#### 1,4,8,11,15,18,22,25-Octa(3-methylbutyl)phthalocyanine (**7e**)

To a flame-dried flask, under an argon atmosphere, lithium metal (0.013 g, 1.86 mmol) was added to a solution of 3,6-bis(3-methylbutyl)phthalonitrile (1.0 g, 3.73 mmol) dissolved in freshly distilled pentanol (10 mL) under reflux. Upon completion of the addition, the reaction mixture turned dark green and was stirred under reflux for 4 h. The mixture was allowed to cool to rt and acetone (5 mL) was added. Excess pentanol was removed in vacuo and the resultant dark green oil, which crystallised on standing, was purified by chromatography on silica gel with 10% DCM/hexane as eluent (*R*_f_ ~ 0.30) to afford the title compound [24] as fine blue-green needles, which were recrystallised from 1:1 THF/acetone (0.81 g, 81%). mp 282–284 °C; DSC (°C, J·g^−1^): Cr 284.1 (40) I 189.9 (−43); IR (neat): 3303 (N–H), 2954 (C–H), 2931 (C–H), 2864 (C–H) cm^−1^; ^1^H NMR (CDCl_3_, 500 MHz) δ 7.86 (s, 8H, C2,3,9,10,16,17,23,24H), 4.46 (t, *J* = 7.5 Hz, 16H, 5 Ar*CH*_2_CH_2_CH(CH_3_)_2_), 1.91 (dt, *J* = 6.8 Hz and 8.1 Hz, 16H, ArCH_2_*CH*_2_CH(CH_3_)_2_), 1.79 (m, *J* = 6.6 Hz, 8H, Ar(CH_2_)_2_*CH*(CH_3_)_2_), 0.99 (d, *J* = 6.6 Hz, 48H, Ar(CH_2_)_2_CH(*CH*_3_)_2_); ^13^C NMR (CDCl_3_, 75 MHz) δ 139.5 (C1,4,8,11,15,22,25), 131.2 (C2,3,9,10,16,17,23,24), 40.4 (C'2), 30.9 (C'1), 27.9 (C'3), 23.4 (C'4); HRMS–ES^+^ *m*/*z*: [M]^+^ calcd for C_72_H_98_N_8_, 1074.7909; found 1074.7857 (100%), 1075.7900 (75%) [M + 1]^+^, 1076.7934 (30%) [M + 2]^+^.

1,4,8,11,15,18,22,25-Octa(4-methylpentyl)phthalocyanine (**7d**)**,** 1,4,8,11,15,18,22,25-octa(5-methylhexyl)phthalocyanine (**7c**), 1,4,8,11,15,18,22,25-octa(6-methylheptyl)phthalocyanine (**7b**) and 1,4,8,11,15,18,22,25-octa((*S*)-3,7-dimethyloctyl)phthalocyanine (**7f**) were all prepared in the same manner (see Supporting Information File 1).

## Supporting Information

Synthesis, analytical and spectroscopic details for the alcohols, alkyl iodides, phthalonitriles **6b–6d** and **6f** and phthalocyanines **7b–7d** and **7f**.

File 1 Experimental details.
