# Supplementary material for: Improved syntheses of high hole mobility phthalocyanines: A case of steric assistance in the cyclo-oligomerisation of phthalonitriles
Source: Beilstein J Org Chem. 2012 Jan 24;8:120–8. doi: 10.3762/bjoc.8.14 (PMC3302073; doi:10.3762/bjoc.8.14)
Supplement: File 1 — Experimental details. [file Beilstein_J_Org_Chem-08-120-s001.pdf]

Supporting Information

for

Improved syntheses of high hole mobility phthalocyanines: A case of steric assistance in the cyclo-oligomerisation of phthalonitriles

Daniel J. Tate[1], Rémi Anémian[2], Richard J. Bushby*[1], Suwat Nanan[1], Stuart L. Warriner[3]and Benjamin J. Whitaker[3]

Address: [1]Centre for Molecular Nanoscience (CMNS), University of Leeds, Woodhouse Lane, Leeds, LS2 9JT, United Kingdom; [2]Merck KGaA, Industriepark Höchst, D 65926 Frankfurt am Main, Germany and [3]School of Chemistry, University of Leeds, Woodhouse Lane, Leeds, LS2 9JT, United Kingdom

Email: Richard J. Bushby - R.J.Bushby@leeds.ac.uk

*Corresponding author

Experimental details

Spectroscopic and analytical details for the alcohols and longer chain alkyl iodides, phthalonitriles and phthalocyanines.

4-Methylpentanol

4-Methylvaleric acid (10 g, 86.1 mmol) dissolved in anhydrous THF (50 mL) was added dropwise to a cooled (0 °C, ice bath) stirred $LiAlH_4$ powder (3.6 g, 94.7 mmol) under an argon atmosphere. Upon completion of the addition the mixture was allowed to warm to rt and stirred for further 2 h. The reaction mixture was carefully poured onto a cooled, stirred saturated solution of Na_2SO_4 (100 mL). The resultant mixture was filtered through a pad of celite to remove inorganic material, which was subsequently washed with diethyl ether (50 mL). The combined organics were extracted into diethyl ether (3 × 50 mL), washed with water (2 × 50 mL), dried ($MgSO_4$) and concentrated in vacuo to afford the title compound as a homogeneous oil (8.3 g, 94%). IR (neat): 3337 (b, OH), 2955 (C–H), 2871 (C–H) cm^{-1}; ^{1}H NMR ($CDCl_3$, 300 MHz) δ 3.65 (t, *J* = 6.6 Hz, 2H, $(CH_3)_2CH(CH_2)_2\mathit{CH_2}OH$), 1.62–1.51 (m, 3H, $(CH_3)_2\mathit{CH}CH_2\mathit{CH_2}CH_2OH$), 1.27–1.19 (m, 2H, $(CH_3)_2CH\mathit{CH_2}(CH_2)_2OH$), 0.89 (d, *J* = 6.6 Hz, 6H, $(\mathit{CH_3})_2CH(CH_2)_3OH$). ^{13}C NMR ($CDCl_3$, 75 MHz) δ 63.7 (C1), 35.2 (C3), 31.0 (C2), 28.2 (C4), 22.9 (C5).

Anal. calcd for $C_6H_{14}O$: C, 70.53; H, 13.81; found: C, 70.60; H, 14.00.

5-Methylhexanol

Prepared by the same method as 4-methylpentanol.

Homogeneous oil (94%). IR (neat): 3338 (b, OH), 2933 (C–H), 2870 (C–H) cm^{-1}; ^{1}H NMR ($CDCl_3$, 300 MHz) δ 3.65 (t, *J* = 7.0 Hz, 2H, $(CH_3)_2CH(CH_2)_3\mathit{CH_2}OH$), 1.60–1.50 (m, 3H, $(CH_3)_2\mathit{CH}(CH_2)_2\mathit{CH_2}CH_2OH$), 1.40–1.28 (m, 2H, $(CH_3)_2CHCH_2\mathit{CH_2}(CH_2)_2OH$), 1.23–1.14 (m, 2H, $(CH_3)_2CH\mathit{CH_2}(CH_2)_3OH$), 0.87 (d, *J* = 6.6 Hz, 6H, $(\mathit{CH_3})_2CH(CH_2)_4OH$); ^{13}C NMR ($CDCl_3$, 75 MHz) δ 63.3 (C1), 38.9

(C4), 33.2 (C2) 28.1 (C5), 23.7 (C3), 22.7 (C6); Anal. calcd for $C_7H_{14}O$: C, 72.35; H, 13.88; found C,72.05; H, 14.10.

6-Methylheptanol

Prepared by the same method as 4-methylpentanol.

Homogeneous oil (92%) IR (neat): 3338 (b, OH) 2954 (C–H), 2930 (C–H), 2869 (C–H) cm^{-1}; ^{1}H NMR ($CDCl_3$, 300 MHz,): δ 3.65 (t, *J* = 6.67 Hz, $(CH_3)_2CH(CH_2)_4\mathit{CH_2}OH$), 1.63–1.47 (m, 3H, $(CH_3)_2\mathit{CH}(CH_2)_5OH$ and $(CH_3)_2CH(CH_2)_3\mathit{CH_2}CH_2OH$), 1.38–1.27 (m, 4H, $(CH_3)_2CH\mathit{CH_2}(CH_2)_4OH$ and $(CH_3)_2CH(CH_2)_2\mathit{CH_2}(CH_2)_2OH$), 1.25–1.13 (m, 2H, $(CH_3)_2CHCH_2\mathit{CH_2}(CH_2)_3OH$), 0.87 (d, *J* = 6.56 Hz, $(\mathit{CH_3})_2CH(CH_2)_5OH$); ^{13}C NMR ($CDCl_3$, 75 MHz) δ 63.12 (C1), 38.95 (C5), 32.85 (C2), 27.92 (C6), 27.19 (C3), 25.99 (C4), 22.64 (C7); HRMS (70 eV, EI): *m/z* (%) 129.1313 (100) $[M]^+$; Anal. calcd for $C_8H_{18}O$: C, 73.78; H, 13.93; found; C, 74.00; H, 14.05.

4-Methylpentyl iodide [1]

Prepared by the same method as 3-methylbutyl iodide.

Homogeneous oil (85%); IR (neat): 2929 (C–H), 2956 (C–H), 2906 (C–H), 2870 (C–H) cm^{-1}; ^{1}H NMR ($CDCl_3$, 300 MHz) δ 3.17 (t, *J* = 7.2 Hz, 2H, $(CH_3)_2CH(CH_2)_2\mathit{CH_2}I$), 1.83 (m, 2H, $(CH_3)_2CHCH_2\mathit{CH_2}CH_2I$), 1.57 (spt., *J* = 6.7 Hz, 1H, $(CH_3)_2\mathit{CH}(CH_2)_3I$), 1.29 (dt, *J* = 6.9 Hz and 5.4 Hz, 2H, $(CH_3)_2CH\mathit{CH_2}(CH_2)_2I$), 0.88 (d, *J* = 6.7 Hz, 6H, $(\mathit{CH_3})_2CH(CH_2)_3I$); ^{13}C NMR ($CDCl_3$, 75 MHz) δ 40.0 (C3), 31.9 (C2), 27.6 (C4), 22.8 (C5), 7.8 (C1); HRMS (70 eV, EI): *m/z* (%) 212.0067 (100) $[M]^+$; Anal. calcd for $C_6H_{13}I$: C, 30.23; H, 5.60; I, 64.08; found: C, 30.40; H, 5.70; I, 63.80.

5-Methylhexyl iodide [2,3]

Prepared by the same method as 3-methylbutyl iodide.

Homogeneous oil (80%); IR (neat): 2955 (C–H), 2931 (C–H), 2869 (C–H) cm^{-1}; ^{1}H NMR ($CDCl_3$, 300 MHz) δ 3.20 (t, *J* = 7.0 Hz, 2H, $(CH_3)_2CH(CH_2)_3\mathit{CH_2}I$), 1.81 (tt, *J* = 7.2 Hz and 7.4 Hz, 2H, $(CH_3)_2CH(CH_2)_2\mathit{CH_2}CH_2I$), 1.55 (spt., *J* = 7.0 Hz, 1H, $(CH_3)_2\mathit{CH}(CH_2)_4I$), 1.39 (m, 2H, $(CH_3)_2CHCH_2\mathit{CH_2}CH_2I$), 1.22 (m, 2H, $(CH_3)_2CH\mathit{CH_2}(CH_2)_3I$), 0.89 (d, *J* = 6.7 Hz, 6H, $(\mathit{CH_3})_2CH(CH_2)_4I$); ^{13}C NMR ($CDCl_3$, 75 MHz) δ 37. 9 (C4), 33.9 (C2), 28. 5 (C3), 27.9 (C5), 22.7 (C6), 7.6 (C1); HRMS (70 eV, EI): *m/z* (%) 226.0226 (100) $[M]^+$; Anal. calcd for $C_7H_{15}I$: C, 37.19; H, 6.69; I, 56.13; found: C, 37.35; H, 6.75; I, 55.85.

6-Methylheptyl iodide [3]

Prepared by the same method as 3-methylbutyl iodide.

Homogeneous oil (84%); IR (neat): 2947 (C–H), 2927 (C–H), 2868 (C–H), 2855 (C–H) cm^{-1}; ^{1}H NMR ($CDCl_3$, 300 MHz) δ 3.19 (t, *J* = 7.17, 2H, $(CH_3)_2CH(CH_2)_4\mathit{CH_2}I$), 1.88–1.77 (m, 2H, $(CH_3)_2CH(CH_2)_3\mathit{CH_2}CH_2I$), 1.58–1.46 (sept, *J* = 6.66 Hz, $(CH_3)_2\mathit{CH}(CH_2)_5I$), 1.43–1.24 (m, 4H, $(CH_3)_2CHCH_2(\mathit{CH_2})_2(CH_2)_2I$), 1.23–1.12 (m, 2H, $(CH_3)_2CH\mathit{CH_2}(CH_2)_4I$), 0.86 (d, *J* = 6.67 Hz, 6H, $(\mathit{CH_3})_2CH(CH_2)_5I$); ^{13}C NMR ($CDCl_3$, 75 MHz) δ 39.01 (C5), 33.87 (C2), 31.04 (C4), 28.17 (C6). 26.60 (C3), 22.89 (C7), 7.75 (C1); HRMS (70 eV, EI): *m/z* Calcd for $C_8H_{17}I$: 240.0375; found, 240.0379 $[M]^+$; Anal. calcd for $C_8H_{17}I$: C, 40.02; H, 7.14; I, 52.85; found: C, 40.25; H, 7.20; I, 52.55.

(*S*)-3,7-Dimethyloctyl iodide [4]

Prepared by the same method as 3-methylbutyl iodide.

Homogeneous oil (86%); IR (neat): 2956 (C–H), 2927 (C–H), 2869 (C–H) cm^{-1}; ^{1}H NMR ($CDCl_3$, 300 MHz) δ 3.27–3.15 (m, 2H, $(CH_3)_2CH(CH_2)_3CH(CH_3)CH_2\mathit{CH_2}I$), 1.94–1.82 (m, 1H, $(CH_3)_2CH(CH_2)_3\mathit{CH}(CH_3)(CH_2)_2I$), 1.70–1.45 (m, 3H, $(CH_3)_2\mathit{CHCH_2}(CH_2)_2CH(CH_3)(CH_2)_2I$), 1.34–1.06 (m, 6H, $(CH_3)_2CH(\mathit{CH_2})_2(CH_3\mathit{CH_2}CH_2I)$, 0.88–0.86 (d, *J* = 6.4 Hz and 6.7 Hz, 9H, $(\mathit{CH_3})_2CH(CH_2)_3(\mathit{CH_3})(CH_2)_3I$); ^{13}C NMR ($CDCl_3$, 75 MHz) δ 41.00 (C2), 39.2 (C6), 36.5 (C4), 33.9 (C7), 27.9 (C3), 24.5 (C5), 22.6 (C7-Me), 18.7 (C3-Me) 5.5 (C1); HRMS (70 eV, EI): *m/z* Calcd for $C_{10}H_{21}I$: 268.0688; found, 268.0684 $[M]^+$; Anal. calcd For $C_{10}H_{21}I$: C, 44.72; H, 7.78; I, 47.32; found C, 44.70; H, H, 8.05; I, 47.30.

3,6-Bis(4-methylpentyl)phthalonitrile (6d)

Prepared by the same method as 3,6-bis(3-methylbutyl)phthalonitrile.

Colourless needles (69%) mp 56–57 °C (petroleum ether); IR (neat): 2952 (C–H), 2928 (C–H), 2871 (C–H), 2226 (CN), 1469 (C=C) cm^{-1}; ^{1}H NMR ($CDCl_3$, 500 MHz) δ 7.47 (s, 2H, C4*H*), 2.83 (t, *J* = 7.9 Hz, 4H, $Ar\mathit{CH_2}(CH_2)_2CH(CH_3)_2$), 1.66 (quin., *J* = 7.9 Hz, 4H, $ArCH_2\mathit{CH_2}CH_2CH(CH_3)_2$), 1.57 (m, *J* = 7.9 Hz, 2H, $Ar(CH_2)_3\mathit{CH}(CH_3)_2$), 1.26 (dt, *J* = 8.8 Hz and 6.7 Hz, 4H, $Ar(CH_2)_2\mathit{CH_2}CH(CH_3)_2$), 0.89 (d, *J* = 6.6 Hz, 12H, $Ar(CH_2)_3CH(\mathit{CH_3})_2$); ^{13}C NMR ($CDCl_3$, 125 MHz) δ 146.6 (C3 and C6), 133.8 (C4 and C6), 116.1 and 115.5 (C1, C2 and CN), 38.7 (C′3), 35.0 (C′1), 29.0 (C′2), 28.2 (C′4), 22.9 (C′5); HRMS (70 eV, EI): *m/z* Calcd for $C_{19}H_{25}N_2$: 281.2018; found, 281.2018 $[M-CH_3]^+$; Anal. calcd for $C_{20}H_{28}N_2$: C, 81.03; H, 9.52; N, 9.45; found: C, 81.05; H, 9.60; N, 9.45.

3,6-Bis(5-methylhexyl)phthalonitrile (6c)

Prepared by the same method as 3,6-bis(3-methylbutyl)phthalonitrile.

Colourless needles (63%) mp 47–48 °C (petroleum ether); IR (neat): 2957 (C–H), 2936 (C–H), 2872 (C–H), 2226 (CN), 1468 (C=C), 1459 (C=C) cm^{-1}; ^{1}H NMR ($CDCl_3$, 500 MHz) δ 7.46 (s, 2H, C4*H* and C5*H*), 2.85 (t, *J* = 7.9 Hz, 4H, Ar$\mathit{CH_2}$$(CH_2)_3CH(CH_3)_2$), 1.63 (quin., *J* = 7.7 Hz, 4H, $ArCH_2$$\mathit{CH_2}$$(CH_2)_2CH(CH_3)_2$), 1.53 (m, *J* = 6.6 Hz, 2H, $Ar(CH_2)_4$*CH*$(CH_3)_2$), 1.36 (quin., *J* = 7.9 Hz, 4H, $Ar(CH_2)_2$$\mathit{CH_2}$$CH_2CH(CH_3)_2$), 1.23 (q, *J* = 6.6 Hz, 4H, $Ar(CH_2)_3$$\mathit{CH_2}$$CH_2CH(CH_3)_2$). 0.86 (d, *J* = 6.6 Hz, 12H, $Ar(CH_2)_4CH($$\mathit{CH_3}$$)_2$); ^{13}C NMR ($CDCl_3$, 125 MHz) δ 146.2 (C3 and C6), 133.4 (C4 and C5), 115. 7 and 115.2 (C1, C2 and CN), 38.6 (C4), 34.5 (C′1), 31.0 (C′2), 27.9 (C′5), 27.0 (C′3), 22.6 (C′6); HRMS (70 eV, EI): *m/z* Calcd for $C_{21}H_{29}N_2$: 309.2331; found, 309.2318 $[M-CH_3]^+$; Anal. calcd for $C_{22}H_{32}N_2$: C, 81.43; H, 9.94; N, 8.63; found: C, 81.40; H, 10.00; N, 8.50.

3,6-Bis(6-methylheptyl)phthalonitrile (6b)

Prepared by the same method as 3,6-bis(3-methylbutyl)phthalonitrile.

Colourless needles (66%) mp 46 °C (petroleum ether); IR (neat): 2928 (C–H), 2864 (C–H), 2227 (CN), 1466 (C=C) cm^{-1}; ^{1}H NMR ($CDCl_3$, 500 MHz) δ 7.47 (s, 2H, C_4*H* and C_5*H*), 2.85 (t, *J* = 7.69 Hz, 4H, Ar$\mathit{CH_2}$$(CH_2)_4CH(CH_3)_2$), 1.70–1.61 (m, 4H, $ArCH_2$$\mathit{CH_2}$$(CH_2)_3CH(CH_3)_2$), 1.58–1.47 (sept, *J* = 6.63 Hz, 2H, $Ar(CH_2)_5$*CH*$(CH_3)_2$), 1.39–1.28 (m, 8H, $Ar(CH_2)_2CH_2(CH_2)_2CH(CH_3)_2$ and $Ar(CH_2)_3$$\mathit{CH_2}$$CH_2CH(CH_3)_2$), 1.20–1.14 (m, 4H, $Ar(CH_2)_4$$\mathit{CH_2}$$CH(CH_3)_2$), 0.86 (d, *J* = 6.63 Hz, 12H, $Ar(CH_2)_5CH($$\mathit{CH_3}$$)_2$); ^{13}C NMR ($CDCl_3$, 125 MHz) δ 146.21 (C3 and C6), 133.43 (C4 and C5), 115.63 and 115.18 (C1, C2 and 2 x CN), 38.78 (C′5), 34.44 (C′1), 30.76 (C′2), 29.43 and 27.07 (C′3 and C′4), 27.92 (C′6), 22.62 (C′7); HRMS (70 eV, EI):

m/z Calcd for $C_{24}H_{36}N_2$: 352.2878; found, 352.2868 $[M]^+$; Anal. calcd for $C_{24}H_{36}N_2$: C, 81.76; H, 10.29; N, 7.95; found: C, 81.45; H, 10.40; N, 7.90.

3,6-Bis((*S*)-3,7-dimethyloctyl)phthalonitrile (6f)

Prepared by the same method as 3,6-bis(3-methylbutyl)phthalonitrile.

Colourless needles (68%) mp 32–34 °C (petroleum ether); IR (neat): 2923 (C–H), 2870 (C–H), 2227 (CN), 1463 (C=C) cm^{-1}; ^1H NMR ($CDCl_3$, 300 MHz) δ 7.46 (s, 2H, C4*H* and C5*H*), 2.89–2.79 (m, 4H, Ar$CH_2$$CH_2$CH($CH_3$)($CH_2$)$_3$CH($CH_3$)$_2$), 0.97 (d, *J* = 6.2 Hz, 6H, Ar(CH_2)$_2$CH(CH_3)(CH_2)$_3$CH(CH_3)$_2$), 0.87 (d, *J* = 6.7 Hz, 12H, Ar(CH_2)$_2$CH(CH_3)(CH_2)$_3$CH(CH_3)$_2$); ^{13}C NMR ($CDCl_3$, 75 MHz) δ 146.5 (C3 and C6), 133.4 (C4 and C5), 115.7 and 115.1 (C1, C2 and CN), 39.2 (C′6), 38.2 (C′2), 36.9 (C′4), 32.7 (C′3), 32.1 (C′1), 28.0 (C′7), 24.7 (C′5), 22.7 and 22.6 (C′8 and C′7-C″1), 19.4 (C′3-C″1); HRMS (70 eV, EI): *m/z* Calcd for $C_{28}H_{44}N_2Na$: 431.3402; found, 341.3380 $[M + Na]^+$; Anal. calcd for $C_{28}H_{44}N_2$: C, 82.29; H, 10.85; N; 6.85; found: C, 82.25; H, 10.85; N, 7.05.

1,4,8,11,15,18,22,25-Octa(4-methylpentyl)phthalocyanine (7d)

Prepared by the same method as 1,4,8,11,15,18,22,25-octa(3-methylbutyl)phthalocyanine.

Fine blue-green needles (62%). mp 232–234 °C (THF/acetone 1:1); DSC (°C, J g^{-1}): Cr 237.1 (43) I 222.6 (−51) Cr; IR (neat): 3295 (N–H), 2948 (C–H), 2934 (C–H), 2867 (C–H) cm^{-1}; ^1H NMR ($CDCl_3$, 300 MHz) δ 7.89 (s, 8H, C2,3,9,10,16,17,23,24*H*), 4.45 (t, *J* = 7.4 Hz, 16H, ArCH_2(CH_2)$_2$CH(CH_3)$_2$), 2.10 (m, 16H, Ar$CH_2$$CH_2$$CH_2$CH($CH_3$)$_2$), 1.61–1.45 (m, 24H, 8 × Ar(CH_2)$_2$$CH_2$*CH*($CH_3$)$_2$), 0.84 (d, *J* = 4.3 Hz, 48H, Ar(CH_2)$_3$CH(CH_3)$_2$); ^{13}C NMR ($CDCl_3$, 75 MHz) δ 138.9

(C1,4,8,11,15,22,25), 130.7 (C2,3,9,10,16,17,23,24), 38.7 (C′2), 33.0 (C′1), 28.3 (C′3), 28.2 (C′4), 22.7 (C′5); HRMS (ES+): *m/z* Calcd for $C_{80}H_{114}N_8$: 1186.9161; found,1186.9126 (100%) $[M]^+$; Anal. calcd for $C_{80}H_{114}N_8$: C, 80.89; H, 9.67; N, 9.43; found: C, 80.65; H, 9.65; N, 9.40.

1,4,8,11,15,18,22,25-Octa(5-methylhexyl)phthalocyanine (7c)

Prepared by the same method as 1,4,8,11,15,18,22,25-octa(3-methylbutyl)phthalocyanine.

Fine blue/green needles (40%). mp 186–187 °C (THF/acetone 1:1); DSC (^{o}C, J g^{-1}): Cr 169.1 (14) Col_h 189.1 (35) I 168.9 (−17) Col_h 161.3 (−15) Cr; IR (neat): 3297(N–H), 2925 (C–H), 2867 (C–H) cm^{-1}; ^{1}H NMR ($CDCl_3$, 500 MHz) δ 7.86 (s, 8H, C2,3,9,10,16,17,23,24*H*), 4.45 (t, *J* = 7.4 Hz, 16H, Ar$\mathit{CH_2}$$(CH_2)_3CH(CH_3)_2$), 2.06 (m, 16H, $ArCH_2$$\mathit{CH_2}$$(CH_2)_2CH(CH_3)_2$), 1.61–1.47 (m, 24H, $Ar(CH_2)_2$$\mathit{CH_2CH}$$(CH_3)_2$), 1.25 (dt, *J* = 6.9 Hz, and 8.7 Hz, 16H, $Ar(CH_2)_2$$\mathit{CH_2}$$CH(CH_3)_2$), 0.79 (d, *J* = 6.7 Hz, 48H, $Ar(CH_2)_3CH($$\mathit{CH_3}$$)_2$); ^{13}C NMR ($CDCl_3$, 75 MHz) δ 138.9 (C1,4,8,11,15,22,25), 130.8 (C2,3,9,10,16,17,23,24), 39.2 (C′2), 32.9 (C′1), 30.9 (C′3), 27.9 (C′5), 27.2 (C′4), 22.6 (C′6); HRMS (ES+): *m/z* Calcd for $C_{88}H_{130}N_8$: 1299.0413; found,1299.0382 (100%) $[M]^+$, 1300.0395 (85%) $[M + 1]^+$, 1301.0441 (40%) $[M + 2]^+$; Anal. calcd for $C_{88}H_{130}N_8$: C, 81.30; H, 10.08; N, 8.62; found: C, 81.15; H, 10.05; N, 8.50.

1,4,8,11,15,18,22,25-Octa-(6-methylheptyl)phthalocyanine (7b)

Prepared by the same method as 1,4,8,11,15,18,22,25-octa(3-methylbutyl)phthalocyanine.

Fine blue/green needles (28%). (THF/acetone 1:1); DSC (°C, J g^{-1}): Cr 112 (15) Cr? 124 (9) Col_h 170 (11) I 162 (−11) Col_h 105 (−9) Cr? 20 (−9) Cr; IR (neat): 3288 (N–H), 2953 (C–H), 2921 (C–H), 2867 (C–H), 1467 (C=C) cm^{-1}; ^{1}H NMR ($CDCl_3$, 500 MHz) δ 7.87 (s, 8H, C2,3,9,10,16,17,23,24*H*), 4.45 (t, *J* = 7.48 Hz, 16H, $Ar\mathit{CH_2}(CH_2)_4CH(CH_3)_2$), 2.07 (m, 16H, $ArCH_2\mathit{CH_2}(CH_2)_4CH(CH_3)_2$), 1.60–1.49 (m, 16 H, $Ar(CH_2)_2\mathit{CH_2}(CH_2)_2CH(CH_3)_2$), 1.48–1.38 (sept, *J* = 6.62 Hz, 8H, Ar $(CH_2)_5\mathit{CH}(CH_3)_2$), 1.36–1.27 (m, 16H, $Ar(CH_2)_3\mathit{CH_2}CH_2CH(CH_3)_2$), 1.14–1.06 (m, 16H, $Ar(CH_2)_4\mathit{CH_2}CH(CH_3)_2$), 0.79 (d, *J* = 6.63 Hz, 48H, $Ar(CH_2)_5CH(\mathit{CH_3})_2$); ^{13}C NMR ($CDCl_3$, 75 MHz) δ 138.82 (C1,4,8,11,15,22,25), 130.78 (C2,3,9,10,16,17,23,24), 38.97 (C′2), 32.86 (C′1), 30.70 (C′3), 29.73 and 27.68 (C′4 and C′5), 27.94 (C′6), 22.58 (C′7); HRMS (ES+): *m/z* Calcd for $C_{96}H_{146}N_8$: 1411.1665; found,1411.1618 (85) $[M]^+$, 1412.1645 (100) $[M + 1]^+$, 11413.1679 (40) $[M + 2]^+$ 1414.1734 (15) $[M + 3]^+$; Anal. calcd for $C_{96}H_{146}N_8$: C, 81.65; H, 10.42; N, 7.93; found: C, 81.55; H, 10.55; N, 7.90.

1,4,8,11,15,18,22,25-Octa((*S*)-3,7-dimethyloctyl)phthalocyanine (7f)

Prepared by the same method as 1,4,8,11,15,18,22,25-octa(3-methylbutyl)phthalocyanine.

Dark green homogeneous oil (83%); IR (neat): 3289(N–H), 2932 (C–H), 2888 (C–H), 2868 (C–H) cm^{-1}; ^{1}H NMR ($CDCl_3$, 500 MHz) δ 7.83 (s, 8H, C2,3,9,10,16,17,23,24*H*), 4.47–4.36 (m, 16H, Ar$CH_2$$CH_2CH(CH_3)(CH_2)_3CH(CH_3)_2$), 2.08–1.99 (m, 8H, $ArCH_2CH_2C\mathit{H}(CH_3)(CH_2)_3CH(CH_3)_2$), 1.78–1.69 (m, 16H, $ArCH_2\mathit{CH_2}CH(CH_3)(CH_2)_3CH(CH_3)_2$), 1.49–1.07 (m, 56H, $ArCH_2CH_2CH(CH_3)\mathit{(CH_2)_3CH}(CH_3)_2$), 0.99 (d, *J* = 6.2 Hz, 24H, $ArCH_2CH_2CH(\mathit{CH_3})(CH_2)_3CH(CH_3)_2$), 0.79 (d, *J* = 6.6 Hz, 48H, $ArCH_2CH_2CH(CH_3)(CH_2)_3CH(C\mathit{H_3})_2$); ^{13}C NMR ($CDCl_3$, 75 MHz) δ 141.2 (C1,4,8,11,15,22,25), 132.9 (C2,3,9,10,16,17,23,24), 41.3 (C′2), 40.3 (C′1), 39.5 , 39.2 , 34.6, 29.9 , 24.6 (C′7-C′1), 24.5 , 22.2; LRMS (FD): *m/z* (%) 1634.3 (80) $[M]^+$, 1635.3 (100) $[M + 1]^+$, 1636.4 (100) $[M + 2]^+$ 1372.4 (80) $[M + 3]^+$; Anal. calcd for $C_{112}H_{162}N_8$ C, 83.01; H, 10.08; N, 6.91; found C, 83.05; H, 10.15; N, 6.85.

References

1. Page, P. C. B.; McKenzie, M. J.; Allin, S. M.; Klair, T. *Tetrahedron* **1997**, *53*, 13149–13164. doi:10.1016/S0040-4020(97)00837-5

2. Berkenbusch, T.; Brueckner, R. *Tetrahedron* **1998**, *54*, 11461–11470. doi:10.1016/S0040-4020(98)00687-5

3. Levene, P. A.; Allen C. H. *J. Biol. Chem.* **1916**, *27*, 443–462.

4. Chen, C.Y.; Nagumo, S.; Akita, H. *Chem. Pharm. Bull.* **1996**, *44*, 2153–2156.
